# Burn or Balm?: Exploring University Students’ Experiences With Social Media During the COVID-19 Pandemic

**DOI:** 10.1177/00332941231175068

**Published:** 2023-05-10

**Authors:** Joanne Lee, Eileen Wood, Natasha Vogel, Edwin Santhosh, Preet K. Chauhan

**Affiliations:** Department of Psychology, 192321 Wilfrid Laurier University, Waterloo, ON, Canada

**Keywords:** COVID-19, social media use, perceived burnout, basic psychological need satisfaction and frustration personality, life satisfaction, Instagram

## Abstract

The impact on perceived burnout experiences among university students from the intensification of social media use during the earliest phase of the COVID-19 pandemic is not yet fully understood. In total, 516 university students (430 females) in a midsized city in Ontario, Canada completed one online survey that explored student characteristics (i.e., personality, life satisfaction, perceived stress, and basic psychological needs) as well as frequency and perceived purpose of social media use. Approximately 80% indicated an increase in their social media use with iMessage/Text messaging, Instagram, and Snapchat being the three most frequently accessed platforms. Social media use was associated with higher levels of perceived stress, extraversion, satisfaction and frustration of psychological relatedness needs, and frustration of competence need. Most students (87%) reported experiencing burnout. Greater burnout was associated with individuals who reported higher perceived stress, scored high in extroversion, and greater use of Instagram. Overall, intensified social media use during the pandemic yielded both positive and negative outcomes.

## Introduction

Social media is a powerful communication tool that is used by millions to make connections with family and friends, work, and school. Within the higher education context, universities and colleges regularly use social media platforms as a means to inform current, potential, and alumni students about events and programming as well as to share reviews related to the institution. In addition, students use multiple platforms to complete educational tasks, gain and share information about courses with their peers and instructors, and stay connected to family and friends (McElroy et al., 2007; Moore & Craciun, 2021; Niu, 2019). Social media use among students, however, saw a significant increase following the sudden and unprecedented shift to online and remote instructional formats following the first wave of COVID-19 in 2020 (Al-Dwaikat et al., 2020; Saud et al., 2020). The impact of increased social media use in this particular context is not yet fully understood. The present study addresses this shortcoming by examining students’ experiences using social media during these challenging times.

Estimates suggest that approximately 65% of adults aged 16 and older significantly increased their use of social media during the first COVID -19 lockdown in 2020 (Bailey et al., 2022; Lemenager et al., 2021). This rise in social media use coincided with the introduction of health measures that limited opportunities for face-to face interactions in all public places including universities/colleges. As a result, many campuses pivoted from in-person instruction to fully online or blended formats with videoconferencing software used for synchronous instructor-student interactions. Given limited means for communicating with peers, instructors and, in some cases, family and friends, it is not surprising that many students turned to social media to connect with others, seek support, and acquire information (Al-Dwaikat et al., 2020; Zhao & Zhou, 2020). The impact of this increased social media use on mental well-being is mixed.

### Social Media Use and Well-Being

Prior to the pandemic, some studies indicated that higher frequency of social media use - the number of times users check for updates or browse profiles and/or the number of social media platforms used - is associated with negative well-being outcomes. For example, heavy social media users aged 19–32 years, who checked their social media platforms more than 58 times per week, tripled their odds of perceived social isolation compared to those who checked fewer than 9 times per week (Primack et al., 2017). Other research findings, including a longitudinal study (van der Velden et al., 2019) and a meta-analysis of 70 articles (Seabrook et al., 2016), report no associations between social media use and mental well-being. During the pandemic, however, some studies have again reported negative impacts associated with increased social media use. For example, greater mental distress, including anxiety and depression, was associated with increased social media use, with especially severe distress levels noted among university students using social media for more than 7 hours per day (e.g., Bailey et al., 2022; Liga et al., 2020). An experimental study suggested a causal link between the amount of screen time spent on social media (i.e., Facebook) and mental distress such as depression and loneliness of university students (Hunt et al., 2018). These detrimental outcomes, however, have not been supported in other research (e.g., Misirlis et al., 2020; Sewall et al., 2022). Inconsistencies in findings both before and during the pandemic underscore the need for further examination regarding the association between social media use and well-being and in particular among students in higher education. The present study extends previous research by exploring individual characteristics of students, their frequency and perceived purpose of social media use to better understand outcomes associated with use and well-being.

Social media encompasses a wide array of platforms, some of which are more diverse in the functions they serve. In addition to frequency of use, the types of activities engaged in while using a platform may be an important consideration for well-being outcomes. Positive associations between social media use and well-being (e.g., increase in life satisfaction and decrease in depressive symptoms) has been shown with active use requiring direct one-on-one communicative exchanges such as sending text messages or broadcasting such as sharing posts to stay connected with family and friends and/or to network with individuals who share hobbies, values, and beliefs (Escobar-Viera et al., 2018; Verduyn et al., 2017). In contrast, passive use of social media platforms without direct communicative exchanges with others (e.g., merely engaging in surveillance of others’ activities, scrolling through news feed or profiles) has a negative impact on well-being if social comparison is experienced by users (Verduyn et al., 2015). Facebook users who engaged in social comparison, for example, experienced more depressive symptoms when they spent more time passively engaged on the platform over multiple logins compared to those who did not (Steers et al., 2014). Surveillance of friends’ activities has also been associated with increased envy (Tandoc et al., 2015). Early reports suggest that similar negative outcomes may be associated with passive social media use among students during the COVID-19 pandemic. For example, university students in Jordan and China experienced an increase in mental distress including anxiety symptoms and negative affect if they used social media platforms for entertainment or accessing COVID-19 news (Al-Dwaikat et al., 2020; Zhao & Zhou, 2020).

Individual differences related to personality traits have been shown to affect the type of social media used. For example, extraverted individuals engage in more active use of social media platforms for communicative exchanges through status updates and comment on friends’ pages on Facebook (Gosling et al., 2011; Kuss & Griffiths, 2011; Ryan & Xenos, 2011) and post on Instagram (Moore & Craciun, 2021) than their introverted peers. On the other hand, individuals with higher levels of neuroticism, characterized by negative affect or emotional instability, spent more time on social media passively such as following friends’ pages (Correa et al., 2010; Ryan & Xenos, 2011) and they do not make more posts or follow more accounts on Instagram than those who are emotionally stable (Moore & Craciun, 2021).

During the pandemic, university students found themselves having to rely predominantly on technologies for their studies, socialization, and work. In Canada, 95.9% of 20- and 24-year-olds identified themselves as regular users of social media and 50.7% reported to have at least three social media accounts in 2018 (Schimmele et al., 2021). Despite being considered digital natives, the intensification of social media use is likely to impact well-being among this group especially with respect to burnout. Hence, the first lockdown restriction offers a unique opportunity to examine why and how social media might contribute to perceived burnout experiences.

Engagement with social media platforms during these challenging times may have fulfilled basic psychological needs. According to self-determination theory (Deci & Ryan, 2000), there are three basic psychological needs for personal growth and positive psychological well-being: autonomy (i.e., the need and freedom to chart one’s life direction that align with one sense of self), competence (i.e., the need to feel adequate and capable of accomplishment), and relatedness (i.e., the need to feel connected to others to have a sense of belonging). Need satisfaction is associated with well-being while need frustration is associated with ill-being (Bartholomew et al., 2011; Gunnell et al., 2014; Vansteenkiste & Ryan, 2013). Accordingly, social media use could facilitate positive well-being outcomes such as life satisfaction if its use *satisfies* these needs. In contrast, its use is most likely to associate with ill-being outcomes such as burnout if these needs are *frustrated* or not being satisfied.

### Present Study

The present study contributes to a growing body of research investigating questions relevant to understanding student use of social media during the COVID-19 pandemic and the relationship use had on perceived well-being. Questions explore the function of social media (e.g., Did social media fulfil gaps ordinarily met in-person?), and the impact of use (e.g., Did social media add to the burden of online and remote work? Did students report burnout?).

Two key areas of inquiry were investigated. First, we examined social media use among university students during the first lockdown of the largest province in Canada during the COVID-19 pandemic. We surveyed frequency of use, social media/networking platforms used, and the purposes students reported for using them. Based on existing research examining use more generally across the pandemic and with broader populations, we expected that Canadian university students would report an increase in social media use during the first lockdown in particular. We further investigated students’ perceptions regarding the function that social media use served at this point in time.

The second area of inquiry concerned burnout as a result of intensified use of social media. Specifically, we examined whether students perceived burnout and what they attributed to the perception of burnout and the role of several individual characteristics (i.e., personality, life satisfaction, and basic psychological needs) that might be associated with burnout.

## Method

### Participants

**Figure 1.**
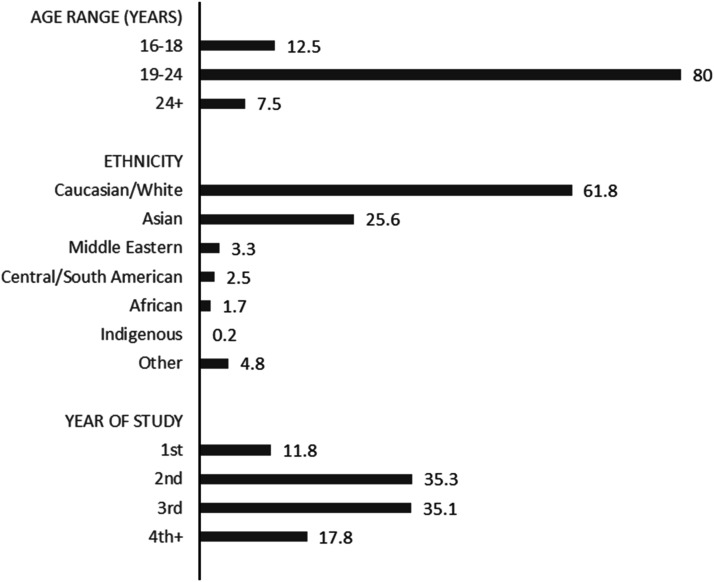
Demographic information of participants reported in percentage.

In total, 565 undergraduate university students (*n*_females_ = 430, *n*_males_ = 79, *n*_other_ = 7) in a mid-sized city in Ontario Canada participated initially, however 49 participants were removed following failure to correctly complete fidelity measures (*n* = 33) and, insufficient survey completion. Ages for the majority of the 516 remaining participants (80%) fell between 19 and 24 years with 12.5% indicating 16–18 years. Most participants (61.8%) self-identified as Caucasian/White, followed by Asian (25.6%). Approximately equal proportions of participants reported being in their second (35.3%) or third year (35.1%) of study. See Figure 1 for detailed demographic information.

Participants were recruited through a university research pool and received course credit for their participation. This project was reviewed and approved by the university’s Research Ethics Board and all participants were treated in accordance with APA/CPA ethical guidelines.

### Materials and Procedure

The study was conducted between June 3 and July 28 of 2020 during the first lockdown of the COVID-19 pandemic. Upon signing up for the study, a link to the online survey was sent and participants completed the 35- to 40-minute survey at their own convenience. The survey assessed demographic information (i.e., gender, age range, year of study, and ethnicity), social media use, life satisfaction, basic psychological needs, perceived stress, personality, and experience with burnout. A copy of the survey questions developed for this study has been submitted to a repository which can be accessed through the following link: https://doi.org/10.5683/SP3/CZZJBI

Participants responded to a forced choice (yes/no/stayed the same) question asking whether their social media use had changed since the beginning of the lockdown in 2020. In addition, participants used a 7-point rating scale (1 = *Rarely or Never* to 7 = *Constantly/Use all the time/Multiple times within an hour throughout the day)*’ to indicate their frequency of social media use for 20 platforms during the preceding month. Participants who reported frequent use of a social media platform (rating of 6 = 3–6 hours per day or more) were subsequently asked to identify all purposes that the social media served for them from a provided list of 4–10 items (e.g., staying connected with family and/or friends, entertainment, school/work, minimize boredom, and learning something new) with the option to identify additional purposes if required.

Life satisfaction was assessed through one question using a 4-point rating scale (1 = *Very dissatisfied* to 4 = *Very satisfied).* Participants also were prompted to explain their rating through an open-ended question.

The 24-item Basic Psychological Need Satisfaction and Frustration Scale (BPNSFS; Deci & Ryan, 2000) comprises six sub-scales associated with self-determination which assess: satisfaction and frustration regarding competence, autonomy, and relatedness needs using a 5-point rating scale (1 = *Not at all true* to 5 = *Completely true*)*.* Reliability for the six subscales was good ranging from α = .70 to α = .87.

The 14-item Cohen’s Perceived Stress Scale (Cohen et al., 1983) assessed participant’s overall perceived stress in the past month using a 5-point rating scale (1 = *Not at all true* to 5 = *Completely true*). The Cronbach’s alpha was high, α = .86.

A brief 10-item version of the Big Five Personality Inventory assessed extraversion, agreeableness, conscientiousness, neuroticism, and openness to experiences (Gosling et al., 2003). Two questions reflected each of the traits and participants rated their level of agreement with each characteristic (e.g., is reserved, quiet) using a 5-point rating scale (1 = *Disagree strongly* to 5 = *Agree strongly*). Reported test-retest reliability = .80 (Gosling, 2003).

Overall burnout from overuse of social media was assessed using one question with a 5-point rating scale (1 = *Never* to 5 = *Very Often*). Participants who indicated some level of burnout (i.e., a rating of two or higher) were provided with an open-ended question asking them to specify the reasons or activities that contributed to feelings of burnout.

#### Coding of open-ended responses

Open-ended responses to questions were coded by question. All responses for each question were read in their entirety by two coders, and coding was initiated on the second reading. The two coders worked collaboratively using an inductive coding strategy to identify emerging themes (Boyatzis, 1998; Strauss & Corbin, 1990). Themes were revised as responses were read. Disagreements were resolved through discussion.

## Results

### Social Media Use

The majority (80%) of participants indicated an increase in their social media use during the first wave of the COVID-19 pandemic, followed by 14.5% identifying no difference and 5.4% reporting a decrease in their social media use.

Of the 20 social media platforms provided, the three most commonly accessed platforms were used for approximately 1–3 hours per day: *iMessage*/*Text messaging* (*M* = 5.18, *SD* = 1.48), *Instagram* (*M* = 5.09, *SD* = 1.44), and *Snapchat* (*M* = 4.71, *SD* = 1.94). An additional two highly used platforms reflecting daily use but less than 1 hour per day included: *E-mail* (*M* = 4.40, *SD* = 1.21) and *YouTube* (*M* = 4.32, *SD* = 1.62), see Table 1. Average use across these five platforms reflected a rating of 1–3 hours use per day (*M* = 4.74, *SD* = .91). The five least commonly used social media platforms (consistent with “Rarely or never” on the 7-point scale) were: *Google Meet* (*M* = 1.07, *SD* = 0.43, *Twitch* (*M* = 1.23, *SD* = 0.87), *Discord* (*M* = 1.41, *SD* = 1.23), *Tinder/Bumble/Hinge* (*M* = 1.43, *SD* = 1.07), and *LinkedIn* (*M* = 1.49, *SD* = 1.0).

**Table 1.** Summary of Mean Ratings for Frequency of Use for Social Media Platforms.

Platform	*M*	*SD*
iMessage/Text messaging	5.18	1.48
Instagram	5.09	1.44
Snapchat	4.71	1.94
Email	4.40	1.21
YouTube	4.32	1.62
TikTok/Douvin	3.76	2.25
Phone calls	3.75	1.42
Facebook messenger	3.55	1.42
FaceTime/Video calls	3.44	1.71
Twitter	3.00	2.03
Zoom	2.30	1.88
Pinterest	2.07	1.39
WhatsApp	1.88	1.48
VSCO	1.86	1.31
Reddit	1.63	1.27
LinkedIn	1.49	1.00
Tinder/Bumble/Hinge	1.43	1.07
Discord	1.41	1.13
Twitch	1.23	0.87
Meetup	1.07	0.43

**Figure 2.**
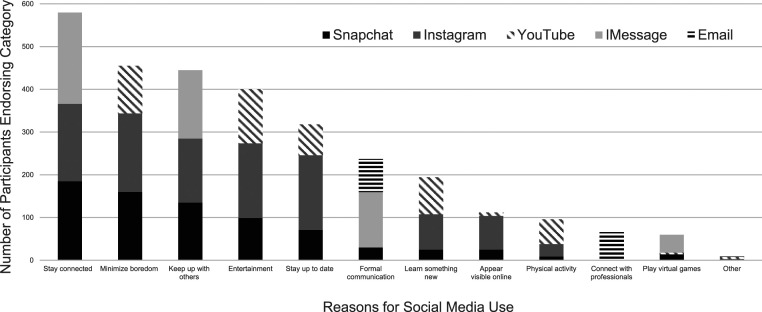
Purposes of social media use for the top five most commonly accessed platforms.

As depicted in Figure 2, the most commonly endorsed reasons for participants who frequently used social media between 3 and 6 hours or more daily for the top five used platforms were: to stay connected, keep up with others, reduce boredom, and for entertainment.

### Life Satisfaction

Participants’ mean ratings of how satisfied they were with their life at present fell close to the ‘Satisfied’ point on the 4-point scale (*M* = 2.85, *SD* = .575). Thematic analysis of the open-ended responses for those who reported positive life satisfaction (satisfied, *n* = 362 or very satisfied, *n* = 44) yielded 15 themes of which two were endorsed by approximately half of the participants: having access to social support (54.4%) and trying to be productive/purposeful (47.5%; see Table 2). The next most frequent theme, being grateful/optimistic/positive, was endorsed by just under a quarter of the participants (21.2%). All other themes were endorsed by less than 18.7% of the participants.

**Table 2.** Summary of Themes Reflecting Reasons for Positive Life Satisfaction Scores.

Theme	*N*	*%*
Having access and support from a social network (family/friends)	221	54.4
Trying to be productive/have purpose in life	193	47.5
Grateful/thankful in general, optimistic, positive outlook	86	21.2
Self-growth, self-reflection, positive reflection, self-awareness, self-care in general	76	18.7
Having a job/employment	70	17.2
Doing enjoyable things	59	14.5
Generally content, not super thrilled but has more of the “it’s alright” or “it’s what it is” attitude	44	10.8
Being healthy	42	10.3
Having the time to do physical activities	37	9.1
Flexibility or in control of daily activities (planning day, online classes whenever)	34	8.4
Good home environment (good living situation, safe)	23	5.7
Financially stable, benefiting financially (e.g., living at home, getting gov. support, etc.)	21	5.2
Spending time outdoors	16	3.9
Other (e.g., got a puppy)	28	6.9

*Note.* Total number of participants = 406 (362 rated Satisfied and 44 rated Very satisfied).

For those who reported negative life satisfaction (dissatisfied, *n* = 101 or very dissatisfied, *n* = 9), coding of these open-ended responses also yielded 15 themes (see Table 3). Four themes were identified by more than 40% of the participants: not feeling productive/purposeful (50%), lacking in-person social connection (42.7%), feeling as if they are not getting ahead in life (42.7%), and not being able to do activities they enjoy (41.8%). All other categories were endorsed by less than 38.2% of the participants.

**Table 3.** Summary of Themes Reflecting Reasons for Negative Life Satisfaction Scores.

Theme	*N*	*%*
Not feeling productive, lost and confused, feeling like they’re not doing enough	55	50.0
Social connection over technology is not fulfilling, missing in-person social connection	47	42.7
Not getting ahead in life, feeling stuck in a position, want to accomplish more	47	42.7
Not being able to do the things that they enjoy, not having access to preferable activities	46	41.8
Academic stress, dislike of school in general	45	40.9
General feelings about the pandemic	42	38.1
Depression, anxiety, loneliness, disconnected, mentioning mental illness, in general	40	36.4
Work stress, financial stress, no paid job	34	30.9
Relationship strains (family, significant others)	34	30.9
Feeling stuck indoors, not being able to go out	31	28.2
Personal physical health issues (knee injury, weight gain), lack of physical activity	17	15.5
Lack of sleep, feeling tired all the time, feeling burnout	12	10.9
Boredom, repetition in their surroundings in terms of social media or their routine or people that they see	10	9.1
Other	31	28.1

*Note.* Total number of participants = 110 (9 rated Very dissatisfied and 101 rated Dissatisfied).

### Basic Psychological Need Satisfaction and Frustration

On average, participants scored above the midpoint of the scale for relatedness satisfaction (*M* = 16.71, *SD* = 2.820), followed by competence satisfaction (*M* = 15.22., *SD* = 3.10), and autonomy satisfaction (*M* = 14.69, *SD* = 2.88). Autonomy frustration (*M* = 11.41, *SD* = 3.27), competence frustration (*M* = 10.65, *SD* = 4.07), and relatedness frustration (*M* = 7.87, *SD* = 3.32) all fell below the midpoint of the scale.

### Personality

Participants on average scored close to the midpoint (maximum = 14) of the scale for extroversion (*M* = 8.57, *SD* = 3.08) and emotional stability (*M* = 8.02, *SD* = 2.81) and slightly higher for agreeableness (*M* = 9.73, *SD* = 2.18), conscientiousness (*M* = 10.73, *SD* = 2.45) and openness to experiences (*M* = 10.50, *SD* = 2.12).

### Perceived Stress

Based on participants’ overall score, the majority of participants (*n* = 411) fell within what Cohen defined as the ‘moderate stress’ category, 87 participants met the ‘low stress’ category and 18 participants fell in the ‘high stress’ group. Although Cohen identified categories, for all analyses using this measure it was treated as a continuous variable (*M* = 38.94, *SD* = 7.12).

### What Influences Social Media Use?

A multiple linear regression was conducted to determine which variables would be associated with social media use. The independent variables included the six subscales of the Basic Psychological Need Satisfaction and Frustration Scale, Cohen’s Perceived Stress Scale, and the five subscales of the Big Five Personality Inventory. The dependent variable was the average use of the top five social media platforms. The overall model was significant, *F* (14, 500) = 4.36, *p* < .001, *R*^*2*^_*adj*._ = .084, with five variables as significant predictors in the model: extraversion (*β* = .13, *p* < .007), relatedness satisfaction (*β* = .21, *p* < .001) and relatedness frustration (β = .14, *p* < .02). In addition, competence satisfaction (β = .04, *p* < .03) and perceived stress (β = .18, *p* < .007) were also significant predictors.

### Burnout and Technology

On average, participants’ ratings of experiencing burnout from the overuse of technology fell between “Sometimes” and “Fairly often” (*M* = 3.35, *SD* = .907) on the 5-point rating scale. There was a positive correlation between the time participants spent on the top five most used social media platforms and burnout, *r* (516) = 0.13, *p* = .004.

Open-ended explanations for the burnout provided by the 448 participants who reported higher levels of burnout (sometimes, *n* = 243; fairly often, *n* = 146; or very often, *n* = 59), yielded 14 themes (see Table 4). As expected, the most common comment indicated the excess use of technology (50%). The next two most frequently identified themes included: boredom due to lack of activities (25.9%), and feelings of being unproductive/lacking purpose (22.3%). All other themes were endorsed by less than 21.2%.

**Table 4.** Summary of Themes for Participants who Indicated Higher Levels of Burnout from the Overuse of Social Media.

Theme	*N*	%
Too much time spent on phone, technology, or social media	224	50.0
Boredom (lack of things to do)	116	25.9
Not feeling productive/lack of purpose/lazy/unmotivated	100	22.3
Quarantine (not being able to go out, feeling stuck indoors, etc.)	95	21.2
Constant negative news and information	79	17.6
Lack of in-person social interaction	78	17.4
Too much screen time causing tiredness, eye strains, headache, losing track of time etc.	76	17.0
Repetition in their surroundings in terms of social media or their routine or people that they seeetc.	65	14.5
Lack of physical activity/lack of movement	61	13.6
Mental illness (depression, anxiety, low self-esteem, stress)	48	10.7
Tired from virtual interactions, tired of talking to others, tired of not having anything new to talk about	46	10.3
Online classes	35	7.8
No routine	20	4.5
Other	40	8.9

*Note.* Total number of participants = 448 (242 rated Sometimes, 146 rated Fairly often, 59 rated Very often).

For those who reported experiencing no burnout (*n* = 13) or minimal burnout (*n* = 55), thematic analysis yielded 10 unique themes that explained these low burnout ratings (see Table 5). The top four of these protective factors included: not excessively using technology (41.2%), having a productive routine (36.8%), being physically active (36.8%), and taking online classes (27.9%). All other factors were identified by less than 26.5% of the sample.

**Table 5.** Summary of Themes for Participants who Indicated Little or No Burnout from the Overuse of Social Media.

Theme	*N*	%
Don’t spend too much time using technology/don’t overuse (phone and social platforms, etc.)	28	41.2
Having a routine, busy doing variety of tasks (chores, spending time with others, baking, etc.)	25	36.8
Physical exercise/staying active/working out with online classes	25	36.8
Online academic classes	19	27.9
Use technology to stay connected	18	26.5
Same amount of technology use before COVID-19, use tech for downtime, relaxing, or in general just enjoy using technology	17	25.0
Going outdoors	17	25.0
Use technology for work	10	14.7
Spending time with family and friends	9	13.2
Other	16	23.5

*Note.* Total number of participants 68 = (13 rated Never and 55 rated Almost Never).

Two correlations were conducted to assess: the relationship between life satisfaction and the extent of burnout as well as the extent of burnout and number of burnout themes endorsed. Overall, there was a negative correlation between burnout and life satisfaction, *r* (516) = −0.23, *p* < .001. There was a moderate positive correlation between the number of burnout reasons participants reported and the extent of burnout experienced, *r* (516) = 0.44, *p* < .001.

### Variables Associated with Burnout

Two multiple linear regressions were conducted to assess variables impacting burnout during the first wave of COVID-19. The first regression was conducted to determine whether burnout was associated with perceived stress, the five subscales of the Big Five Personality Inventory, the six subscales of the Basic Psychological Need Satisfaction and Frustration Scales, and life satisfaction. The model was significant, *F* (13, 501) = 8.88, *p* < .001, *R*^*2*^_*adj.*_ = .17. Two variables predicted burnout: perceived stress (*β* = .34, *p* < .001) and extroversion (*β* = .09, *p* < .04) such that higher stress and higher levels of extroversion predicted greater burnout (See Table 6).

**Table 6.** Standardized Coefficients for Perceived Stress, Big Five Personality traits, Basic Psychological Needs, and Life Satisfaction on Burnout Frequency.

Predictor variable	B	Std. Error	Beta	Significance (*p*)
Perceived stress	.043	.008	.340	.001**
Extroversion	.026	.013	.090	.042*
Agreeableness	−.010	.018	−.024	.574
Conscientiousness	.003	.017	.008	.863
Emotional stability	.001	.016	.004	.938
Openness	.031	.019	.072	.108
Autonomy satisfaction	.014	.019	.044	.456
Autonomy frustration	.013	.015	.046	.385
Relatedness satisfaction	.013	.019	.040	.487
Relatedness frustration	.003	.016	.012	.838
Competence satisfaction	−.036	.019	−.123	.064
Competence frustration	−.003	.015	−.013	.847
Life satisfaction	−.088	.080	−.056	.274

*p < .05. **p < .001.

The second regression was conducted to determine whether time spent on each of the five most frequently reported social media platforms predicted burnout. The model was significant, *F* (5, 510) = 2.46, *p* < .032, *R*^*2*^_*adj.*_ = .014. As shown in Table 7, only use of Instagram predicted burnout (*β* = .12, *p* = .031) such that greater time spent on Instagram predicted greater burnout.

**Table 7.** Time Spent on the Five Most Frequently Used Social Media Platforms on Burnout Frequency.

Predictor variable	B	Std. Error	Beta	Significance (*p*)
Instagram	.073	.034	.116	.031*
Snapchat	.000	.024	.001	.987
IMessage	.013	.032	.021	.693
Email	−.004	.035	−.005	.914
YouTube	.045	.025	.081	.073

*p < .05.

## Discussion

The primary goal of the present study was to determine how social media use impacted students lives during the first lockdown of the COVID-19 pandemic. A growing body of research has identified negative mental health impacts (e.g., high stress, anxiety, and depression) and increased reliance and use of technologies associated with the pandemic (Bailey et al., 2022; Li et al., 2021). Pivoting to online formats was a necessity for many both in their work and learning contexts. However, the technologies and programs necessary to shift to remote and online communications for work and study did not necessarily require a similarly high degree of shift to social media platforms in particular. Our study examined the use of social media platforms separately from other technologies that were a required part of the online pivot during the pandemic. Although social media use is a pervasive part of the lives of young adults in general (Perrin & Andersen, 2019; Schimmele et al., 2021), our study indicates that use of social media became even greater for the vast majority of the students during the COVID-19 pandemic lockdown.

Consistent with extant literature (Schimmele et al., 2021), students in the present study engaged in multiple social media platforms with average of approximately 11 platforms accessed. However, a more limited number of platforms was accessed for the highest frequency of use. Our findings suggest that use of platforms varied both in terms of amount of use and the function that the platform served. Five platforms were identified as those most heavily used among these students. Four of these platforms (i.e., iMessage/Text messaging, Instagram, Snapchat, and YouTube) overlapped in their function with all providing a means for students to keep up to date. In addition to keeping students informed, the top three platforms (i.e., iMessage/Text messaging, Instagram, and Snapchat) also served to keep students connected with others. Further, Snapchat, Instagram, and YouTube, were identified as tools that helped to minimize boredom and provided a source of entertainment. Across a full array of 20 platforms, use patterns make clear that students do not rely on a single source to meet their goals. Instead, the majority of students utilized a small number of platforms to achieve goals related to news, engagement with others, and entertainment.

An important role served by social media platforms was communication. Specifically, students identified iMessage/Text messaging, Snapchat and email as tools for formal communication. Interestingly, email, which was the fourth most prevalent platform used, was the only platform identified for use in communications with professionals in particular. This may be a reflection of the primary means of communication between students and their instructors. Within the university context, many instructors and universities encourage use of university specific email accounts for security reasons (Duran et al., 2005). Similarly, many online learning management systems have email tools which permit direct communication with instructors. Thus, it is not surprising that email was identified as the tool used to communicate with professionals. iMessage/Text messaging and Snapchat, on the other hand, may have been a more common tool for formal communication with others beyond the university setting, including employers. Together, the shared and unique uses of platforms demonstrate discrimination among social platform tools in terms of perceived effectiveness for attaining different goals. Students were selective, goals were diverse, and students met their goals by selecting specific platforms to meet each goal.

Use of social media was associated with individual characteristics. Specifically, consistent with expectations and previous research (Amichai-Hamburger & Vinitzky, 2010; Kraut et al., 2002; Moore & Craciun, 2021), individuals higher in extroversion were more likely to use social media. This is most likely due to their need to be present and engaged with others. Given the limited outlets for engagement during the lockdown (e.g., no in-person meetings, no extracurricular activities, no restaurants, limited shopping), it is not surprising that those high in extroversion would seek social connections and stimulation through social media outlets.

Individuals reporting greater stress during this time period were also more likely to use social media. Literature documents the time of the pandemic as a stressful time for many (Brooks et al., 2020) with many more individuals in general reporting higher levels of stress (Kowal et al., 2020; Salari et al., 2020). Some students also reported greater stress during the pandemic (Al-Dwaikat et al., 2020; Elmer et al., 2020). With few outlets available for relief students may have been more likely to engage in greater levels of social media use as a means to alleviate stress through, seeking out information, consultation, and engaging in discussion groups related to academic pursuits (Al-Dwaikat et al., 2020; McElroy et al., 2007), connecting with others (Elmer et al., 2020; Prowse et al., 2021), entertainment (Perugini & Solano, 2021), exercise (Burke & Rains, 2019; Saud et al., 2020). Social media would be one of the few sources available to serve as a conduit for achieving these goals.

Three subscales associated with self-determination (Deci & Ryan, 2000) were associated with higher social media use. Specifically, competence satisfaction was associated with greater social media use. Higher levels of competence satisfaction align with greater confidence in one’s ability to achieve goals effectively (Liga et al., 2020). Students may have used social media as a means to achieve goals. For example, engaging social media platforms to connect with other students to study, to complete assignments, and to receive information about courses (Al-Dwaikat et al., 2020; Khan et al., 2021) or to create content or posts for others to share their knowledge or promote themselves (Moore & Craciun, 2021; Sheldon & Bryant, 2016).

Interestingly, both relatedness satisfaction and relatedness frustration were associated with greater social media use. Relatedness generally, refers to one’s need to establish relationships, close social connections, and to be part of a community. It is not surprising that many of the functions students identified as important for their social media use involve being connected to others, whether that involved staying in touch, staying up to date and connected with the events of the world, or communication (with professionals, or generally). Satisfaction of relatedness need is associated with well-being (Vansteenkiste & Ryan, 2013). The positive relationship between satisfaction of relatedness need and increased social media use suggests that, for some students, greater social media use would be associated with a greater sense of well-being. However, it is also the case that frustration of relatedness need, which is associated with ill-being, was associated with greater social media use. Although this may appear contradictory, this finding may provide some explanation for conflicting evidence currently reported in the literature (e.g., Bailey et al., 2022; Liga et al., 2020; Misirlis et al., 2020; Sewall et al., 2022) where both positive and negative outcomes have been associated with increased social media use. On the surface, it may appear that our findings are reflecting the experiences of two unique groups: one that had positive outcomes through their use of social media and one that had negative outcomes. However, in this study it appears that greater use of social media can result in both relatedness satisfaction and frustration. Both are possible, as the use of social media may have served as a mechanism for reaching out to others, establishing oneself within their communities, and satisfying the more basic need to be with others. However, limitations inherent in the use of online connections as the sole or primary means of connecting with others may have failed to achieve some of the needs ordinarily achieved through more direct, in-person contact. Individuals may have felt separated from others and perceived increasing social isolation (Primack et al., 2017; Shensa et al., 2016), and technology simply could not suffice to meet that need, resulting in higher levels of relatedness frustration. In addition, the way in which social media was used may also contribute to relatedness satisfaction and frustration. Specifically, active use of social media platforms where users actively initiate and engage in communicative exchanges to stay connected with family and friends as well as to network with individuals with shared interests and values (Escobar-Viera et al., 2018; Verduyn et al., 2017) may be more likely to result in positive social outcomes. High levels of passive use such as following friends’ Facebook pages or following celebrities’ Instagram accounts (Ryan & Xenos, 2011; Verduyn et al., 2015) or consuming COVID-19 pandemic news (Wathelet et al., 2020), however, have been associated with negative social outcomes. Thus, although the uses of social media platforms appear to serve important functions, and greater social media use may have facilitated some social needs, it also may have failed to meet some critical social needs especially if its use were for surveillance purposes such as following celebrities’ pages or posts (Hill & Zheng, 2018).

In terms of other possible outcomes associated with high use of social media, the vast majority of students in our study also experienced some level of burnout (including: being drained of physical or emotional energy, loss of motivation, being tired from virtual interaction, easily irritated, frustrated with daily routine, and feeling unproductive). Unlike existing literature which has examined burnout more generally (academic burnout, Jiang, 2021; work exhaustion, Oksanen et al., 2021), what is new in the present study is that in this case burnout was reported as specific to the overuse of social media platforms. Specifically, 87% of the students reported experiencing burnout at or above the midpoint of the scale (sometimes or more) with 46% of these students endorsing the two highest levels of the burnout scale. Two individual characteristics which predicted greater social media use also predicted those most likely to experience burnout from overuse of social media. Not surprisingly, individuals higher in extroversion were more likely experience higher levels of burnout. As noted above, filling the void left by typical sources of engagement through higher use of social media may have led to excessive use and greater burnout. In addition, high levels of perceived stress were associated with greater burnout. Consistent with a considerable body of research (Brooks et al., 2020; Fofana et al., 2020), increased stress was an artifact of the pandemic for the general public especially during a lockdown. This increased stress was noted among students in particular (Bailey et al., 2022; Zhao & Zhou, 2020). Continuous messaging about threats to health, risks regarding exposure, and concerns regarding unemployment, closures etc. Communicated through many social media platforms (Abd-Alrazaq et al., 2020; Tsao et al., 2021; Yigitcanlar et al., 2020) combined with the constant reminder that individuals were socially distant and apart from others likely contributed to feelings associated with burnout. The emotional challenges of navigating the early stressors of the pandemic are well documented (Brooks et al., 2020; Salari et al., 2020; Zhang et al., 2020). Such stressors were likely an important contributor to emotional fatigue and burnout associated with overuse of social media for students.

The relationship of specific platforms to burnout yielded only one social media platform of concern. Overuse of Instagram in particular, the second most highly endorsed social media platform used, was associated with burnout. Instagram allows individuals, including celebrities, to highlight the best moments or interests in their lives through pictures and videos in real time. Instagram users may be accessing the platform constantly to view these posts for fear of missing out (FOMO), defined “as a pervasive apprehension that others might be having rewarding experiences from which one is absent” (Przybylski et al., 2013, p. 1841). Instagram users with higher FOMO have been found to be heavy users of social media and follow more Instagram accounts than those with lower FOMO (Moore & Craciun, 2021). During the pandemic and, in particular, during lockdowns when students may have felt isolated, cut off from others and limited in their own activities and goals, they may have turned to outlets such as Instagram to feel connected and engaged. However, social comparisons resulting from increased use of Instagram may have intensified aspects of burnout such as frustrations with daily routines, feeling unproductive, and being tired from virtual interaction, thus resulting in greater perception of burnout. In addition, given that students’ own lives would be much more limited during the pandemic, their use of Instagram may have been more passive than active and passive use of social media platforms is associated with poor mental well-being (Steers et al., 2014; Verduyn et al., 2015). Also, students may have turned to Instagram to see what others were doing, but have little to add themselves, thus exacerbating negative social comparisons.

### Practical Implications

The dramatic shift to online only instruction as part of the lockdown associated with the pandemic continues to have repercussions today as universities return to the new ‘normal’. The shift revealed that students could be taught at a distance, that online options were possible for a wide range of courses across academic levels and disciplines, and that there were potential economic advantages for students and institutions for online versus in-person instruction. Studies such as the present research are an important contribution when considering potential advantages and disadvantages associated with instructional delivery. The present study clearly indicates that such conversations should take into consideration the role and impact of social media use when students perceive themselves to be distant or isolated which may occur when little choice is available in terms of course delivery methods.

To date a great deal of literature regarding social media focuses on affordances and challenges associated with the use, misuse, and access to social media in terms of communication and social interaction issues. The present study extends these considerations and highlights the importance of understanding social media use when considering public health policy specifically with respect to mental and physical health. For example, our findings underscore the importance of informing regular social media users about potential pitfalls with the use of these platforms, especially those that inherently involve more passive use and how use has implications for physical activity and nutrition. Users also should be cognizant of how these platforms could elicit negative social comparison, envy, and FOMO, all of which are associated with mental health concerns such as emotional fatigue and perceived stress. Hence, it is timely to extend our understanding of outcomes associated with social media use to policy considerations, especially those targeting general well-being, and mental health.

### Limitations

The present study provides a snapshot of students’ experiences during the unique occurrence of the first lockdowns and imposed restrictions associated with the global COVID-19 pandemic. Burnout in the present study is based on student self-reports. Although corroborating physical measures (e.g., software analytics, tracking, health measures) may have been a useful addition to the study, the rapidity with which the study needed to be executed and limitations in types of measures that could be used due to restrictions in contact prohibited use of other measures. However, the self-report method employed in the present study does provide information regarding students’ perceptions of their experiences during this unprecedented event.

## Conclusions

Previous literature has yielded conflicting outcomes regarding variables related to well-being and ill being (e.g., anxiety, depression, etc.). Our findings help to explain why these conflicting findings have been found. Overall, high use of social media during the pandemic yielded both positive and negative outcomes. In some cases, increased social media use served a protective factor allowing students to gain information, connect with others, and engage in productive, health-related and entertainment-based activities. However, very high reliance also was a risk factor associated with burnout, as students predominantly relied on social media to fill the void of boredom due to the lack of activities they typically engaged in during the COVID-19 lockdown.
